# Dura sac compression due to spinal epidural gas pseudocyst after lumbar decompression surgery: a case report

**DOI:** 10.1186/s12891-019-2682-1

**Published:** 2019-06-21

**Authors:** Jianwei Guo, Xuexiao Ma, Yong Liu, Guanghui Li, Dexun Wang, Zhongying Wang, Shuzhong Li

**Affiliations:** Department of Spine Surgery, The Affiliated Hospital of Qingdao University, 16 Jiangsu Road, Qingdao, 266003 Shandong Province People’s Republic of China

**Keywords:** Lumbar surgery, Dura sac compression, Gas pseudocyst

## Abstract

**Background:**

Intraspinal gas pseudocyst is rare, especially following spinal surgery. Here we present a case of spinal epidural gas pseudocyst following lumbar decompression surgery, which caused dura sac compression.

**Case presentation:**

A 52-years-old woman with chronic lumbar pain and radiating numbness of left leg was admitted to our hospital and underwent a posterior lumbar decompression surgery. 10 days later, the patient began to have dysfunction of excretion. CT and MRI were taken and epidural gas was detected, which compressed the dura sac. A huge pseudocyst encapsulated with high-tension air was found during debridement with no evidence of infection.

**Results:**

Debridement surgery was taken to remove the encapsulated gas and cyst wall and her symptoms disappeared soon after the surgery. 2 weeks later, routine X-ray was repeated and gas pseudocyst disappeared with no signs of infection.

**Conclusion:**

Gas pseudocyst in the spinal canal is rare, especially after lumbar surgery and causing spinal cord compression. CT and MRI can be used to detect the spinal gas. Once gas pseudocyst causes dura sac compression, proper methods should be chosen to treat this kind of intraspinal gas pseudocyst.

## Background

Intraspinal gas was first reported in the intervertebral degenerative disc of the spine by Magnusson. With the use of computerized tomography (CT), intraspinal gas was becoming apparent not only in the degenerative spinal disease [1–5], but also in tumor [6, 7], infection [6–8], spinal trauma [9] and iatrogenic processes [5, 10, 11]. However, it is very rare that intraspinal gas causes dura sac compression and becomes symptomatic, especially after spinal surgery. Here we present a case of gas pseudocyst following lumbar decompression surgery, which caused spinal cord compression.

## Case presentation

**Fig. 1:**
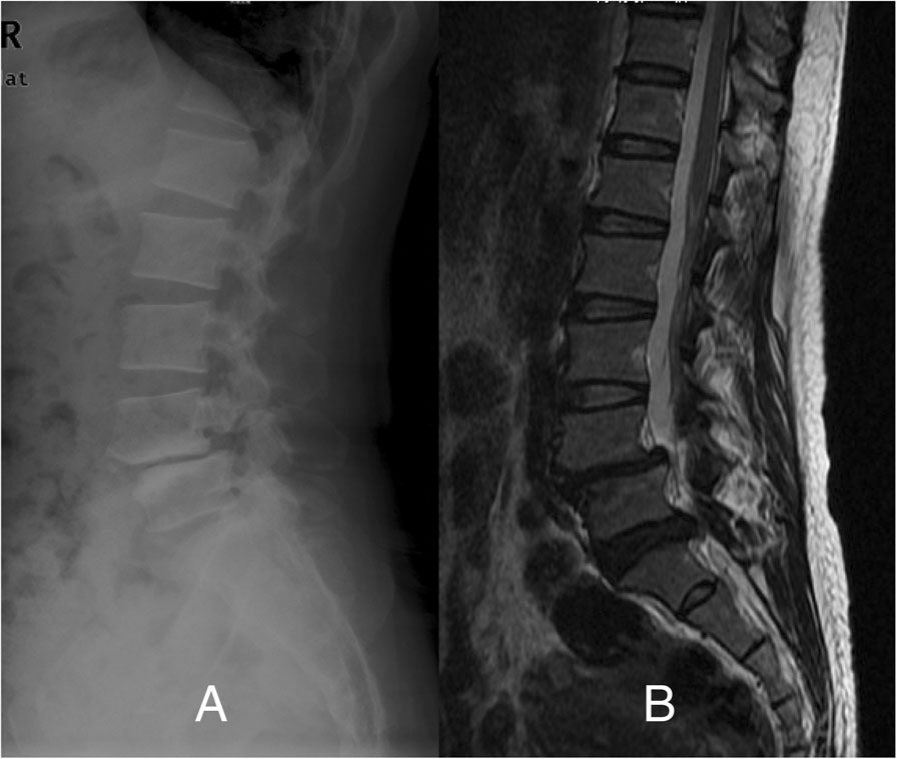
Preoperative lateral X-ray (**a**) and sagittal MRI (**b**) of lumbar spine demonstrated L4 spondylolisthesis and spondylolysis in both sides and disc protrusion of L5/S1 compressing left L5 and S1 root

A 52-year-old woman was admitted to our hospital, suffering from chronic lumbar pain for ten years and aggravating after physical labour. 4 months ago, she experienced violent low back pain and numbness of left leg, radiating from left hip to the low extremity with no obvious predisposing cause. Physical examination showed tenderness of interspinal areas in the low lumbar and hypesthesia to pinprick in the L5 and S1 dermatomal distribution, as well as positive straight leg raising sign. Routine spinal X-rays showed L4 spondylolisthesis and spondylolysis in both sides. CT scan reconfirmed L4 spondylolisthesis and spondylolysis in both sides and showed evidence of disc protrusion and lumbar stenosis at L4/5 and L5/S1, compressing left L5 and S1 root (Fig. 1).

**Fig. 2:**
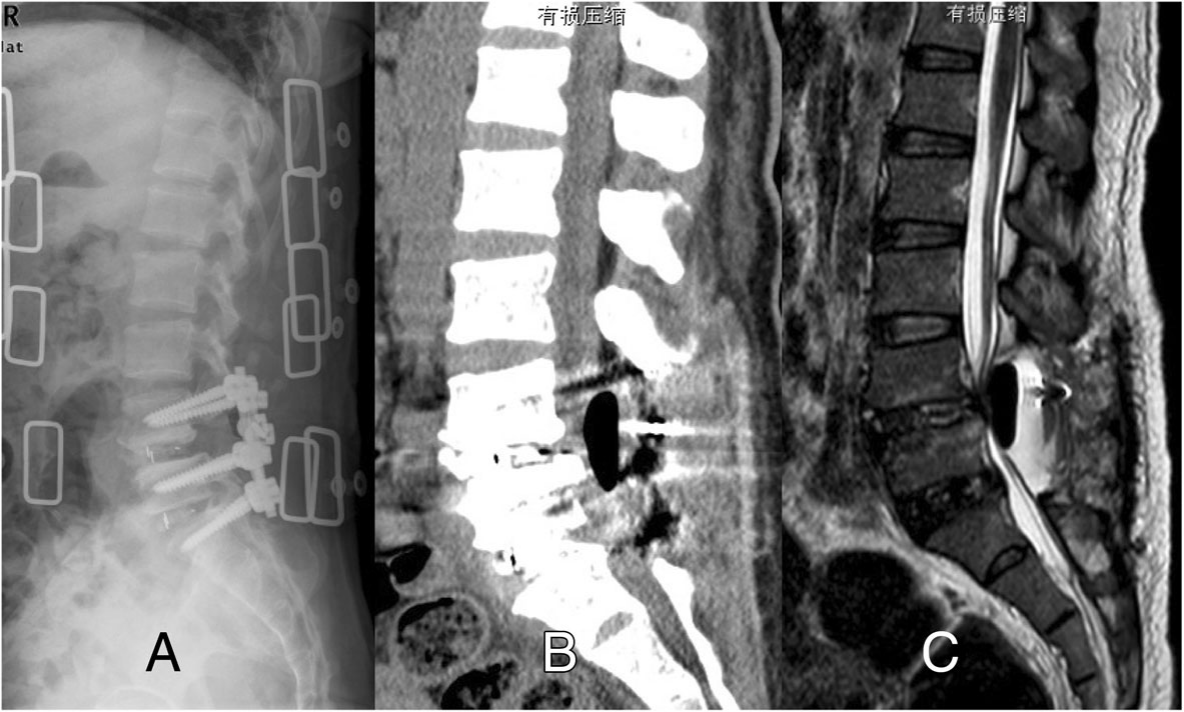
Postoperative lateral X-ray of lumbar spine after surgery revealed a black shadow in the upper surgical region (**a**). The patient had no comfort and no intervention was taken. 10 days later, she began to have dysfunction of excretion. CT and MRI (**b** and **c**) revealed a collection of epidural gas pseudocyst, compressing the spinal cord

**Fig. 3:**
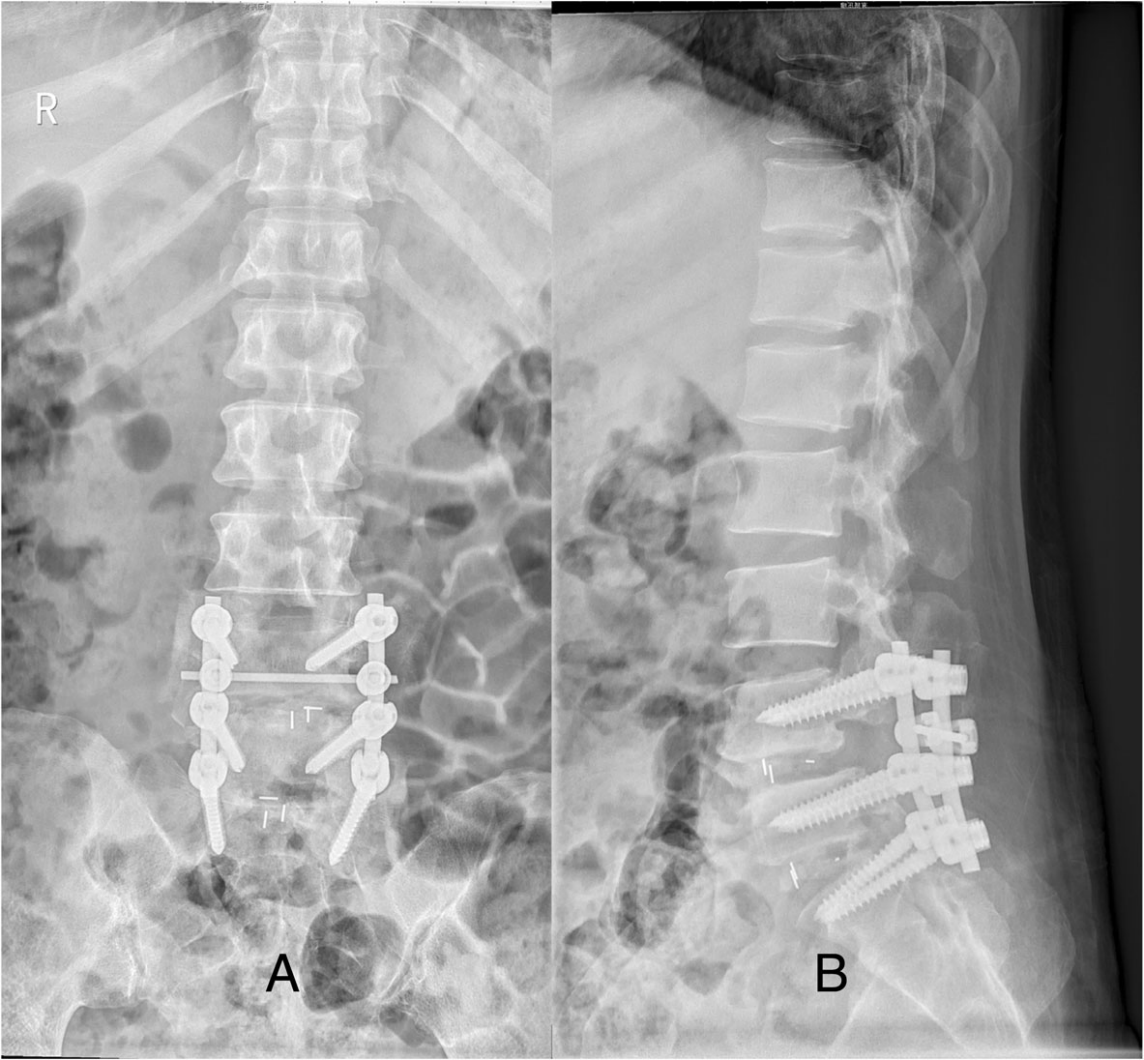
Postoperative lumbar X-ray after debridement (**a** and **b**) showed gas pseudocyst disappeared with no signs of infection

The patient subsequently took a posterior lumbar decompression surgery at L4-S1, internal fixation and intervertebral fusion at L4/5 and L5/S1. Satisfied outcome was achieved and the patient got out of our hospital at the 6th day. Routine X-ray test was taken before her discharge and showed internal fixations were in good places, but with a black shadow at the surgical place. 10 days later, the patient had an uncontrolled lumbar sprain and began to have dysfunction of excretion. She was readmitted to our hospital. CT and MRI were taken and gas pseudocyst was detected, which compressed the dura sac (Fig. 2). Laboratory studies before the revision surgery were taken to rule out the possibility of infection. Debridement surgery was taken. During the surgical procedure, a sound of rushing air was heard when the deep fascia and muscle were opened through the previous route. A thin, blister-like membranous structure surrounded with little clot organization were seen in the surgical field, significant compressing the dural sac with no sign of infection. The membranous structure and clot organization were removed until dura sac were decompressed and returned to throb. Isotonic saline and diluted iodophor solution were used to irrigate surgical field and intradiscal space at the last stage of the operation to prevent persistence of air and infection in spinal canal. Her symptoms disappeared soon after the surgery. Routine X-rays were repeated 2 weeks after debridement, and gas gap disappeared with no signs of infection (Fig. 3).

## Discussion

The presence of spinal gas within an intervertebral disc or spinal canal can be seen at vertebral spondylosis [4, 12, 13], vertebral osteomyelitis [7, 14], vertebral metastasis [6, 7], vertebral trauma [9], after iatrogenic manipulation [5, 10], after thoracotomy [11, 15, 16], or Kummel disease. Spinal gas was first found in the intervertebral soft tissue of spine by Magnusson in 1937, called vacuum disc phenomenon. This so-called vacuum disc phenomenon is believed to be associated with degenerative disc disease. With the degeneration of intervertebral disc, fissuring of the fibrocartilage occurs in the desiccated nucleus pulposus and collects gas dissolved in the extracellular fluid under subatmospheric pressure [17]. This kind of gas contains more than 90% of nitrogen, which cannot be reabsorbed or replaced by liquid because of no vascular in the degenerative disc [17].

Gas collection in the spinal canal is believed to have the similar mechanism with gas collection in the degenerative disc. Gas may be originated from the venous circulation and subsequent diffuse into the vertebral canal [15, 16]. Gas in the spinal canal, in the nerve root foramina [18], in the epidural space [13, 19], in the subarachnoid space [20, 21] or in a spinal epidural cyst [22, 23] may cause spinal cord or root compression and lead to severe neurological symptoms, which may need surgical treatment.

As one possible cause of intraspinal gas, intraspinal gas following spinal surgery is common. Most of postoperative intraspinal gas is asymptomatic and re-absorbed in a few weeks. Only a few cases of symptomatic intraspinal gas have been reported in previous reports (Table 1) [24–29].

**Table 1:** Features of intraspinal gas pseudocyst in previous reports

Reference	Age(yr)/sex	Primary operation	Reoccurred symptoms	Imaging findings (CT or MRI)	Treatment
Raynor and Saint-Louis [24], 1999	35/M	L4/5 disc excision	a foot drop and pain in the same leg 15 days later	a gas bubble located at the right L5 lateral recess	Conservative treatment
Kaymaz et al. [25]., 2005	NR	L4/5 discectomy and foraminotomy	weakness in dorsal flexion on the contra lateral leg	air trapping within the L3/4 epidural space	Conservative treatment
Capelle and Krauss [26], 2006	50/F	Removal of L5/S1 free herniated disc without discectomy	recurrent pain in same leg 4 days later	epidural gas formation at the operative site	Failure of conservative treatment for 7 days, re-operation
Ilica et al. [27], 2006	44/M	L4/5 disc excision	lower back pain and SLR 45° + 5 months later	accumulation of gas in the lumbar epidural space	Failure of conservative therapy for 1 months, re-operated after no response
Sasani et al. [28], 2007	62/F	Microdiscectomy without foraminotomy or hemilaminectomy	recurrent pain 20 days later	air bubble in the right anterolateral portion of the L2/3 epidural space	Conservative treatment
	72/F	Microdiscectomy and stabilization	recurred pain in left leg 2 weeks later	gas collection on the L4/5 left mediolateral	Failure of conservative therapy for 3 days and needle aspiration for 2 days; surgery successful
	69/M	L5/S1 microdiscectomy and foraminotomy	radiculopathy in his right leg 7 days later	gas bubble in the L5/S1 epidural space and disc space	Conservative treatment
Chul-Woo, et al. [29], 2014	68/F	L2/3 and L5/S1’laminectomy and discectomy	lower back pain and radiating pain to her left leg similar to preoperative symptoms 2 weeks later	air accumulation compressing L3/4 and L5/S1 dural sac and nerve root	Failure of conservative treatment for 2 weeks and needle aspiration for 2 days; surgery successful

The exact origin and pathogenetic mechanisms of postoperative intraspinal gas is still unknown. A few hypotheses were introduced to explain the origin and possible pathogenic mechanisms of gas accumulation in the spinal canal [28]. Some authors believed that postoperative intraspinal gas might be originated from trapped gas within the soft tissue during the operation, intradiscal gas, or spinal structure itself [28, 29]. There may be the existence of communication between interspinal gas and intradiscal gas. Gas may migrate from operated and adjacent disc to the epidural space through this communication under the normal movements of lumbar spine, just acting as a piston. Finally, intraspinal gas accumulated and increased, leading to dura sac or nerve root compression [13]. What’s more, the usage of spinal instrument may provide gap for air accumulation between spinal bone structure and muscle, especially the presence of cross connection.

Different methods have been introduced to treat postoperative intraspinal gas. For asymptomatic intraspinal gas, conservative treatment may be a good choice. Absolute bed rest and immobilization with brace to restrict postoperative lumbar spine motion are recommended to lower the chances of air re-accumulation. Percutaneous needle aspiration had been used to treat asymptomatic intraspinal gas [30]. However, the outcomes of this method are controversial. Some authors reported that intraspinal air re-accumulated and previous symptom re-occurred after percutaneous needle aspiration because of piston-like mechanism and presence of membrane encasing air [29]. Open surgery is recommended in case of no response to conservative treatment or failure of percutaneous needle aspiration. Cyst walls, the gaseous cyst, and the membranous soft tissue near the nerve root during the operation must be removed in order to prevent re-accumulation or persistence of air in the lumbar epidural space. Due to the great advances in endoscopic techniques and equipment, percutaneous endoscopic treatment for the symptomatic epidural gas-containing pseudocyst is also a good choice with sufficient decompression, low recurrence rate, and minimal invasion [31]. Besides, irrigating the surgical field with isotonic saline and longer stay of the drain postoperatively may be helpful to prevent the formation and increase of intraspinal air.

## Conclusion

It is rare that intraspinal gas following spinal surgery causes spinal cord compression. Intraspinal gas may accumulate and become asymptomatic under normal movement of lumbar spine segments. More attention should be paid to this kind of intraspinal gas pseudocyst in case of neurologic symptoms. Proper methods should be chosen to treat this kind of intraspinal gas pseudocyst.

## Data Availability

All the raw data is contained within the manuscript and additional files.

## Abbreviations

CT: Computed tomography
MRI: Magnetic Resonance Imaging
